# Imaging features of skeletal muscle lymphoma: a case report and literature review

**DOI:** 10.1186/s12880-021-00667-4

**Published:** 2021-09-26

**Authors:** Shuxi Gao, Hong Shu, Hua Yang

**Affiliations:** 1 Department of Ultrasound, Shengjing Hospital of China Medical University, Shenyang, 110004 Liaoning Province Republic of China; 2 Department of Pathology, Shengjing Hospital of China Medical University, Shenyang, Liaoning Province Republic of China

**Keywords:** Lymphoma, Skeletal muscle, Ultrasound, Biopsy, Imaging, Case report

## Abstract

**Background:**

Diffuse large B cell lymphoma (DLBCL) is the most common type of non-Hodgkin lymphoma (NHL), occurring predominantly in older people. Skeletal muscle lymphoma is a rare form of DLBCL, most frequently affecting the thigh, upper extremities, calf, and pelvis.

**Case presentation:**

We report a case of skeletal muscle DLBCL that was diagnosed using ultrasound (US)-guided biopsy. A 70-year-old man presented with progressive swelling and pain in the left lower extremity and an elevated erythrocyte sedimentation rate (ESR) and serum C-reactive protein (CRP), ferritin, and CA125 levels. US, magnetic resonance imaging (MRI), and computed tomography (CT) showed diffuse lesions in several muscles of the left lower extremity. Positron emission tomography/CT (PET/CT) showed FDG-uptake in the affected muscles. The patient was treated with chemotherapy and achieved a good response. A systematic review of the literature published between 1992 and 2019 was conducted to investigate the role of imaging, including imaging-guided biopsy, in the diagnosis of skeletal muscle lymphoma.

**Conclusions:**

Skeletal muscle lymphoma is rare. US and MRI features include enlargement of muscular structures, with preservation of the architecture of the tissue and surrounding anatomical structures. Definitive diagnosis relies on histological and immunohistological analysis of a sample obtained through imaging-guided biopsy.

## Background

Lymphoma is a heterogeneous group of malignancies of lympho-reticular origin. Lymphomas can arise anywhere in the body and are traditionally divided into Hodgkin and non-Hodgkin lymphoma (NHL). Extranodal lymphoma involves sites other than lymph nodes, including the gastrointestinal tract, lung, central nervous system, salivary glands, thyroid, and gonads [1]. Extranodal lymphoma involving the musculoskeletal system, including bone, cutaneous/subcutaneous tissue and muscle, is rare [2]. The reported frequency of muscle lymphoma accounts for 0.1% to 1.4% of all extranodal lymphomas and 1.2–2.0% of all malignant muscle tumors [3].

NHL is classified into B cell or T cell lymphomas. Diffuse large B cell lymphoma (DLBCL) is the most common type of NHL, occurring predominantly in older people [4]. Skeletal muscle lymphoma is a rare form of DLBCL, most frequently affecting the thigh, upper extremities, calf, and pelvis [2]. Patients with skeletal muscle lymphoma usually present with a progressively enlarging mass, swelling, pain, fever, sweating, and weight loss [5].

Imaging modalities, including computed tomography (CT), ultrasound (US) and magnetic resonance imaging (MRI), have been utilized to evaluate skeletal muscle lymphoma, and imaging characteristics of skeletal muscle lymphoma have been reported in small case series and case reports. However, diagnosis based on imaging criteria alone is not conclusive, as imaging features of skeletal muscle lymphoma are variable.

Image-guided biopsies provide a safe and effective method for the conclusive diagnosis of musculoskeletal tumors [6]. US is frequently used as the initial imaging modality for evaluating a superficial symptomatic mass, and percutaneous US-guided biopsy is a convenient, radiation-free, and relatively inexpensive choice for biopsy of musculoskeletal soft tissue lesions [7]. Here, we report a case of skeletal muscle DLBCL of the lower extremity that was diagnosed by histological and immunohistological analysis of a sample obtained through percutaneous US-guided biopsy. We conducted a systematic review of the literature published between 1992 and 2019 to investigate the role of imaging, including imaging-guided biopsy, in the diagnosis of skeletal muscle lymphoma.

## Case presentation

A 70-year-old Chinese male was admitted to the General Surgery Department of Shengjing Hospital (an affiliate of China Medical University) due to a 3 month history of fever and progressive swelling and pain in the left lower extremity in the absence of trauma or infection. The patient attended his local hospital when the symptoms began. There, a Color Doppler US examination showed dilated deep and intramuscular veins of the left lower extremity, with slow blood flow; therefore, intramuscular venous thrombosis of the calf was suspected. US examination revealed decreased echogenicity of the muscles of the medial and posterior compartments of the left thigh and the muscles of the posterior compartment of the calf, with a diffuse increase in vascularity. Blood tests showed an elevated D-dimer level, white blood cells were 8.04 × 10^9^/l, monocytes were 11.90%, neutrophils were 76.30%, red blood cells were 3.61 × 1012/l, and hemoglobin was 95 g/L. The diagnosis was suspected left calf intramuscular venous thrombosis, possibly due to cellulitis. The patient was given anticoagulation and parenteral antibiotics, including 800,000 units penicillin G sodium qd (i.v), 110 mg dabigatran ester bid (i.v), an antipyretic, and nutritional support. The swelling, pain and fever worsened, with a maximum temperature of 42 °C.

**Fig. 1:**
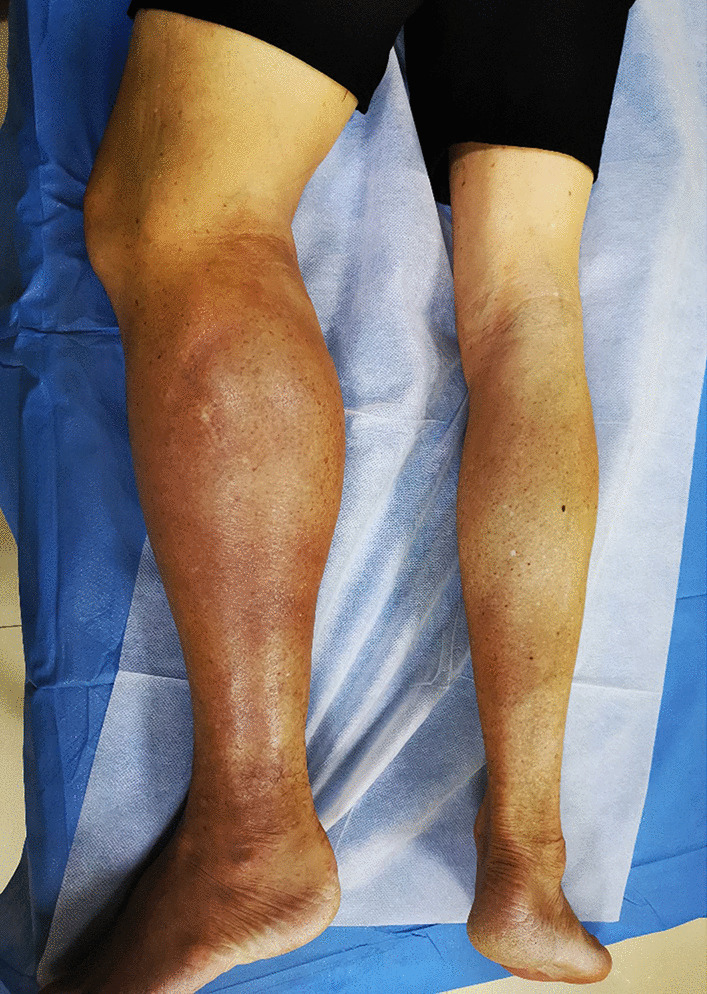
The left lower extremity was obviously swollen, and the skin was dark red in color

The patient was transferred to our institution. He had no relevant medical or family history, and he reported no history of weight loss or change in appetite. General physical examination was unremarkable. There were no signs of hepatosplenomegaly or abdominal, respiratory or cardiovascular disorders. On clinical examination, a hard, non tender, ill-defined mass was present in the left inguinal region. The left lower extremity was obviously swollen and tender, and the skin was dark red in color and warm to touch (Fig. 1). Neurological examination was normal. Routine blood and liver function tests were normal. Laboratory tests showed serum C-reactive protein (CRP) was 292.0 mg/L (normal 0–8), erythrocyte sedimentation rate (ESR) was 58 mm/h (normal 0–15), ferritin was 993.7 ng/ml (normal 11–336.2), CA125 was 66.32 U/mL (normal 0–35), β_2_-microglobulin (b2-MG) was 9.42 mg/L (normal 0.7–1.8), and lactic acid dehydrogenase (LDH) was normal. IgG and IgA levels were decreased.

**Fig. 2:**
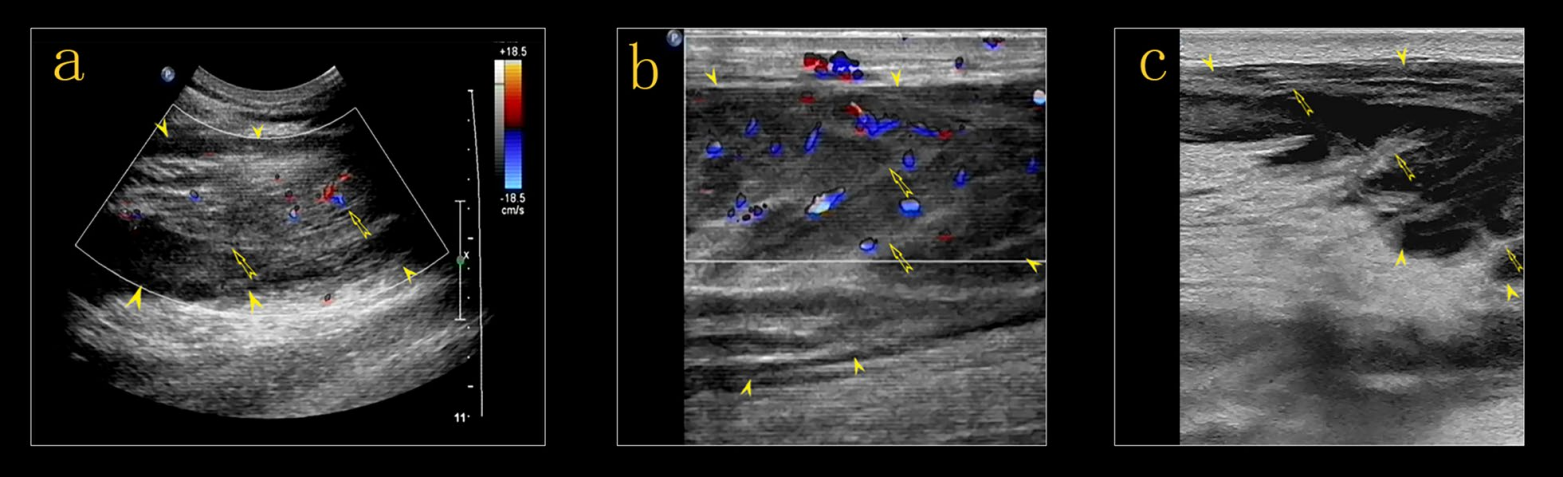
Ultrasound examination of the left lower extremity revealed large poorly defined hypoechoic regions (*arrow head*) within the muscles of the medial compartment of the thigh (**a**) and gastrocnemius (**b**, **c**). The hypoechoic regions irregularly infiltrated normal muscle tissue, with texture resembling muscle fibers retaining continuity with the surrounding muscles *(arrow)*. The architecture of the adjacent muscles appeared to be preserved. Color and power Doppler ultrasound showed hypervascularity (**a**, **b**)

US examination of the left lower extremity revealed large ill-defined hypoechoic regions within the muscles of the medial and posterior compartments of the thigh and the muscles of the posterior compartment of the calf, which were diffusely swollen, with preservation of the underlying muscle architecture. The regions were located at the margin or on one side of the muscles, irregularly infiltrating normal muscle tissue, with texture resembling muscle fibers retaining continuity with the surrounding muscles. Adjacent anatomical structures and tissues preserved their normal architecture. Color and power Doppler US showed hypervascularity within the regions (Fig. 2), but no evidence of thrombosis of the deep or superficial veins of the left lower limb. An enlarged lymph node was detected in the left inguinal area. On ultrasound, the inguinal lymph node had a thick hypoechoic periphery, a hyperechoic central region, and a clear distinction of the border of the cortex and medulla. Lymph node vascularity was increased and had a branch-like distribution. Increased echogenicity in the subcutaneous tissues indicated edema. The provisional diagnosis was an intramuscular lesion.

**Fig. 3:**
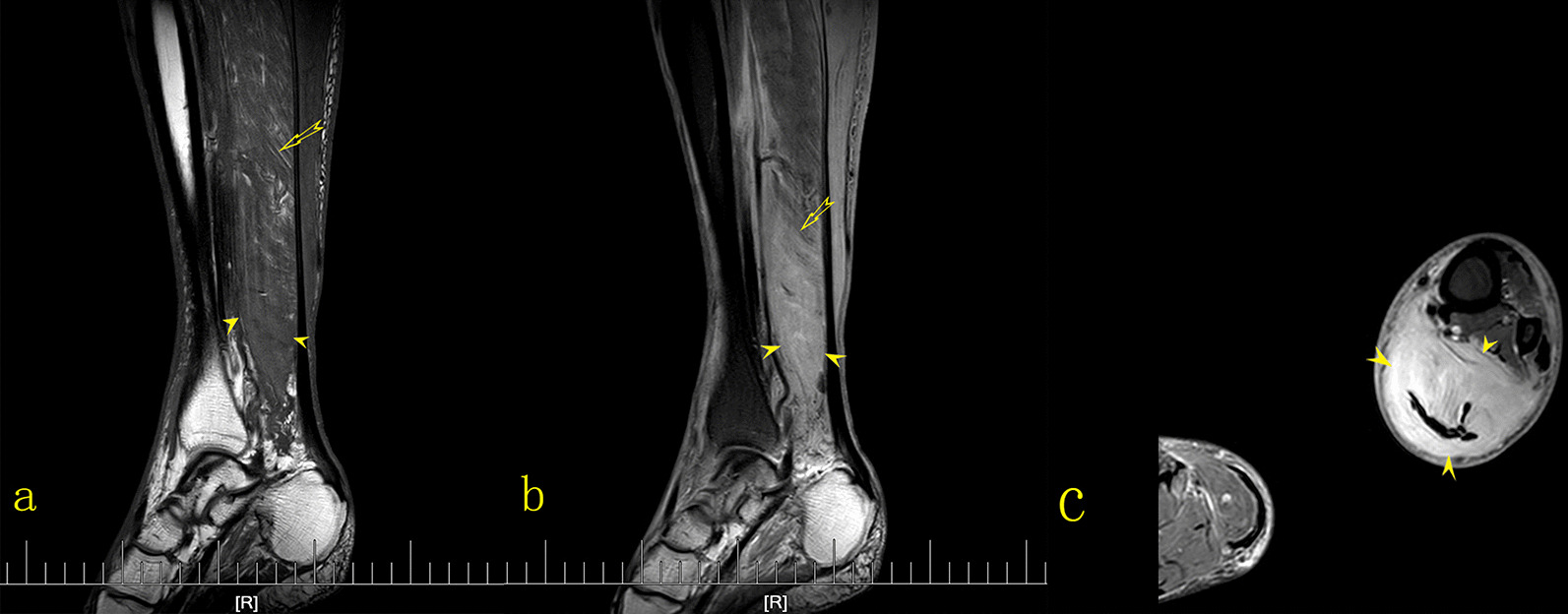
Sagittal T1-weighted images (**a**), T2-weighted images with fat suppression (**b**), and axial T2-weighted images with fat suppression (**c**) showing diffuse swelling that infiltrated the gastrocnemius (*arrow head*), a minimally heterogeneous hypointense signal on T1-weighted images, a hyperintense signal on T2-weighted sequences (*arrow* in **a**, **b**), and an ill-defined boundary

**Fig. 4:**
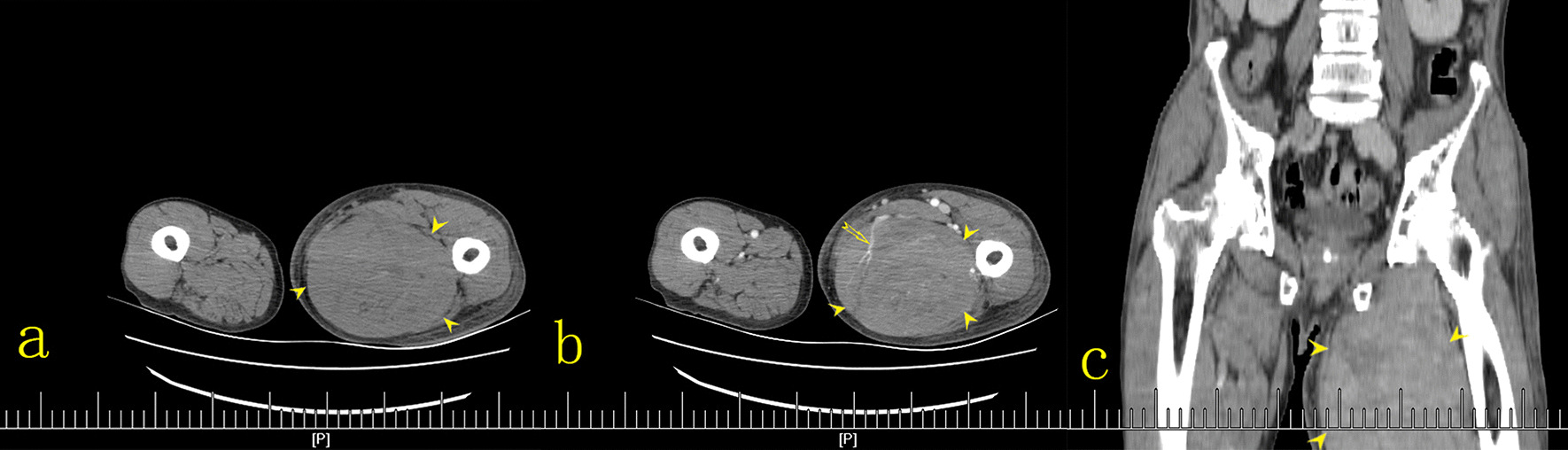
Axial plain (**a**), intravenous-contrast-enhanced (**b**), and coronal reconstruction-contrast-enhanced (**c**) CT scans showing diffuse swelling of the muscles of the medial compartment and posterior compartment of the thigh. Muscles were enlarged and contained patchy hypodense regions, with indistinct margins (*arrow head*). The vessels enhanced as they normally would and probably appeared more conspicuous as compartmental compression impairs blood flow (*arrow* in **b**). Following intravenous contrast administration, there was slightly patchy and mild enhancement of the lesions (**b**, **c**)

Subsequently, MRI and CT were performed. Contrast-enhanced CT was used to evaluate the pelvic cavity and lower abdominal cavity, while MRI was used to evaluate the lower leg lesions. MRI of both calves showed diffuse swelling that infiltrated the muscles of the posterior compartment of the left calf. The distal aspect of the gastrocnemius and soleus had a minimally heterogeneous hypointense signal on T1-weighted images and a hyperintense signal on T2-weighted and fat suppression sequences (Fig. 3), with an ill-defined margin. Muscle fibers could be seen in some lesions, but they were indistinct. CT scans were performed on the lower abdomen and bilateral thighs. Non-contrast CT showed diffuse swelling of the muscles of the medial and posterior compartments of the thigh. The muscles were enlarged, and contained patchy hypodense regions, with indistinct margins. Fat planes between muscles were lost. After intravenous contrast administration, enhancement of the involved muscles was slightly patchy, and mild. Attenuation after contrast administration did not appear to be substantially different from other muscle groups when compared to the contralateral side or the quadriceps muscle or gluteal bellies on the same side. The vessels enhanced as they normally would and probably appeared more conspicuous as compartmental compression impairs blood flow (Fig. 4). Abdominal and chest CT scans were normal.

**Fig. 5:**
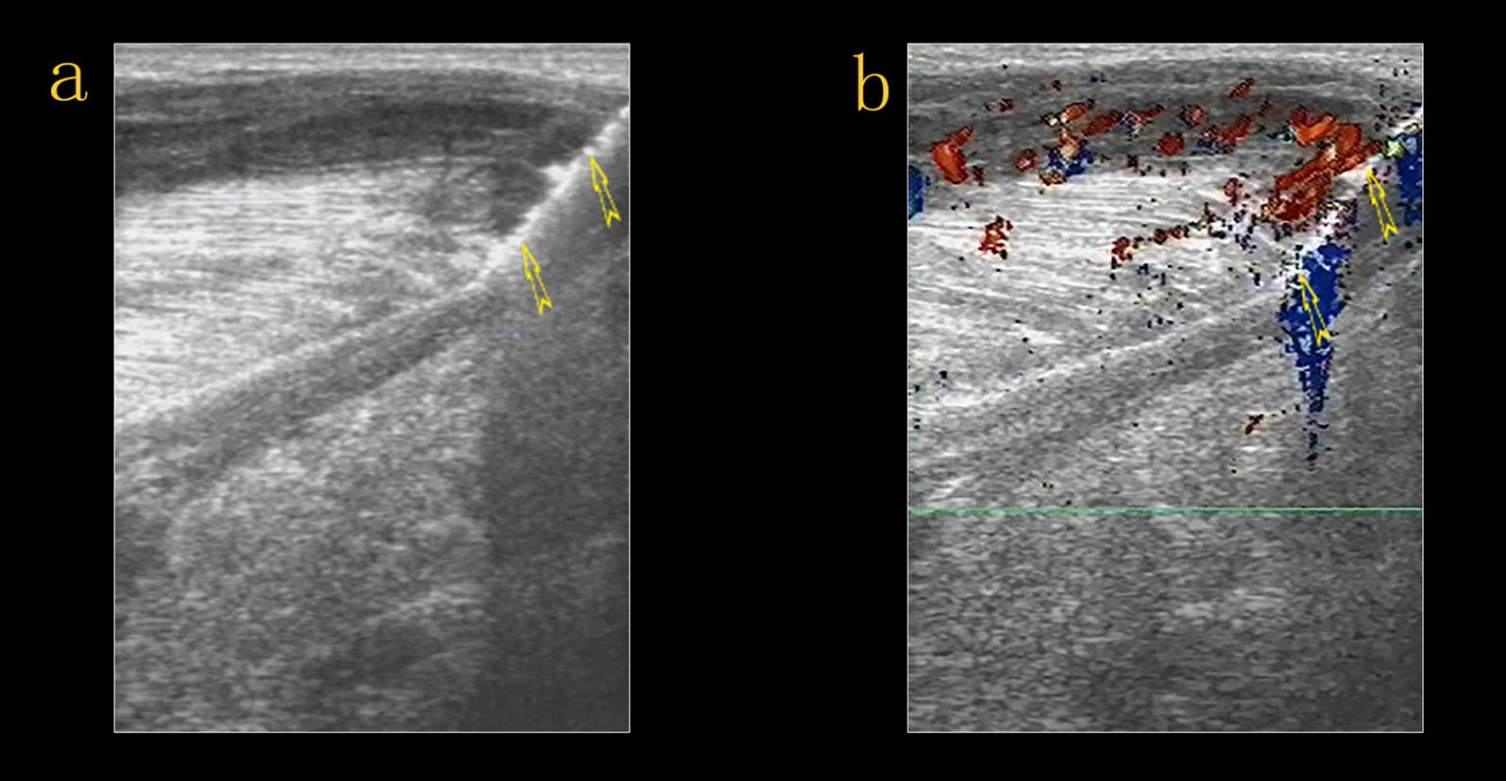
Percutaneous US-guided biopsy of the hypoechoic solid-appearing parts of the lesions within the gastrocnemius (*arrow*)

The patient was provisionally diagnosed with intramuscular lesions; however, it is challenging to base a pathological diagnosis on imaging characteristics alone. Extensive patchy lesions and elevated inflammatory markers were consistent with inflammation, while a tumor that irregularly infiltrated the muscle suggested a different diagnosis, necessitating imaging-guided biopsy. Consequently, percutaneous US-guided core needle biopsy was performed under local anesthesia (2% lidocaine) using a 16 gauge needle. The hypoechoic solid-appearing parts of the regions within the gastrocnemius were targeted, while large blood vessels and nerves were avoided (Fig. 5). Four cores were obtained after four passes, without any immediate complications.

**Fig. 6:**
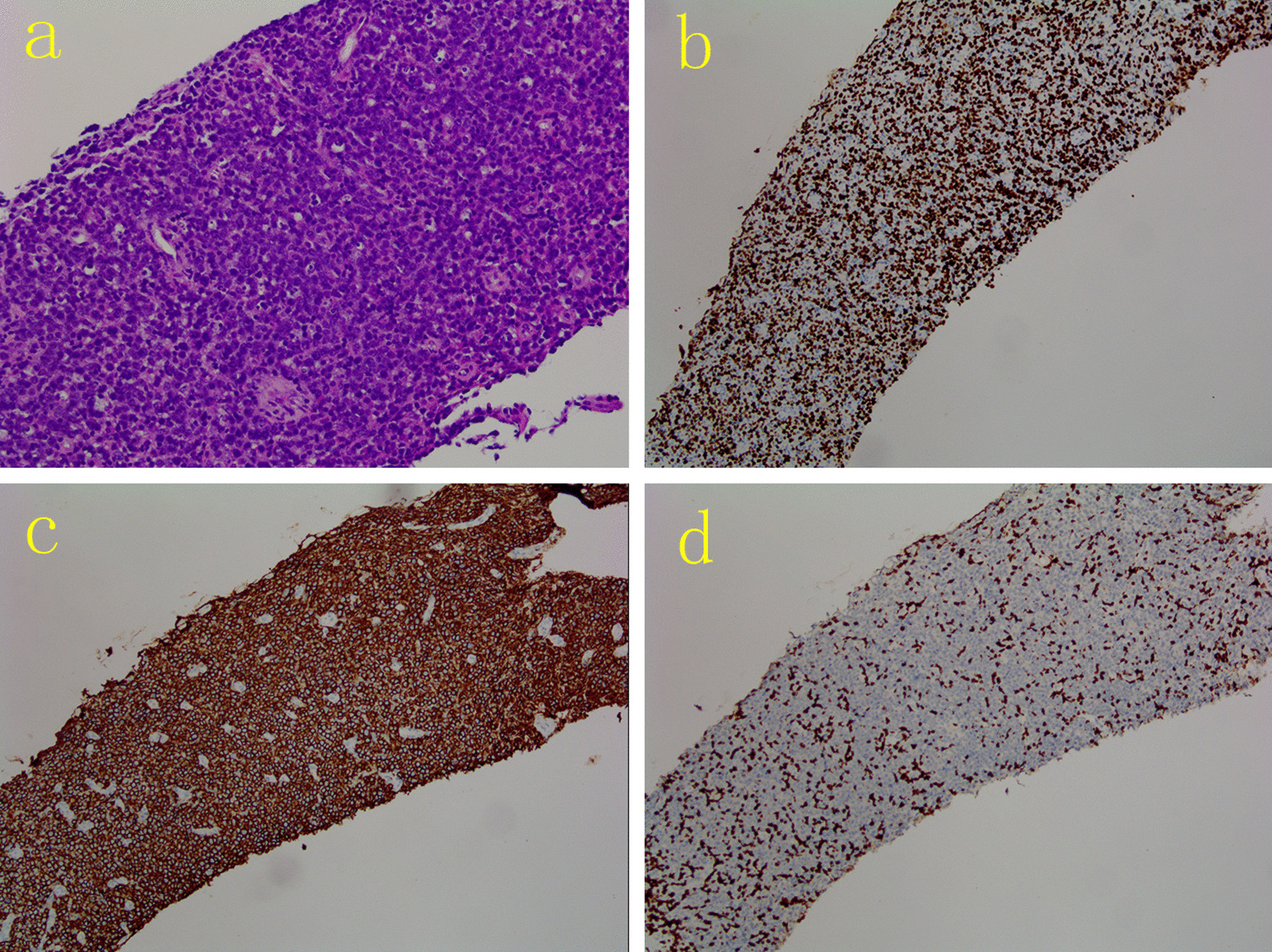
Histopathological findings (**a**; hematoxylin–eosin [H&E] staining, 200 ×) showing medium sized tumor cells arranged in diffuse sheets, and immunohistochemical (IHC) staining (**b**–**d**; 100 ×) showing the Ki-67 proliferation index was 90% (**b**), and tumor cells were positive for CD20 (**c**) and negative for CD3 (**d**)

Biopsy specimens were fixed in 10% buffered formalin, processed, and embedded in paraffin. Microscopic examination revealed small round lymphoid cells arranged in diffuse sheets. On immunohistochemistry, tumor cells were positive for CD20, CD19, vimentin, Bcl-2, Bcl-6 and MUM-1, scattered positive for CD5, cyclin D1 and P53 protein, and negative for CK, CD21, CD23, CD3, CD10 and desmin. The Ki-67 proliferation index was 90%. In situ hybridization was positive for Epstein-Barr encoded RNAs (EBER). The histopathological diagnosis was non-Hodgkin DLBCL (Fig. 6).

**Fig. 7:**
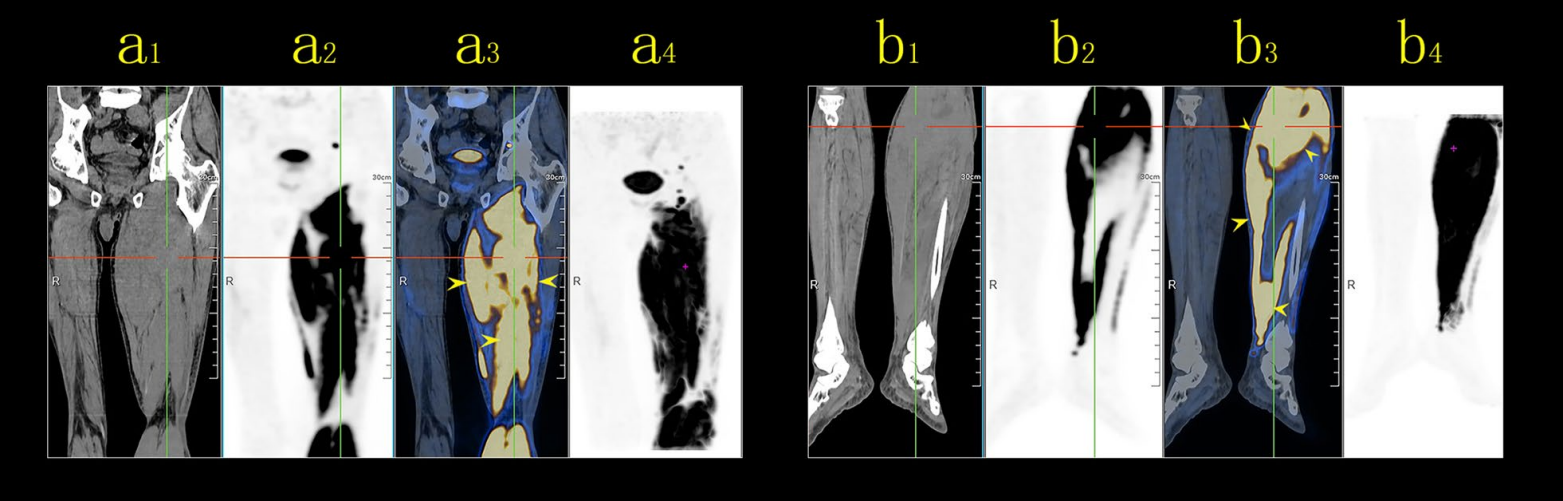
Positron emission tomography/computed tomography (PET/CT) showing multiple lesions of increased FDG-uptake in the left obturator externus muscle, the muscles of the medial and posterior compartments of the thigh (**a**), and the posterior and peroneal muscles of the calf (**b**). (a1 &b1) CT image showing ill-defined intramuscular lesions. (a3 & b3) PET/CT image showing multiple lesions with FDG uptake (maximum standardized uptake: 10.20). (a2, a4 and b2, b4) FDG images

A staging whole body fluorodeoxyglucose (FDG)-positron emission tomography (PET) was performed to identify other sites of disease. F18-fluorodeoxyglucose PET/CT (F18-FDG PET/CT) demonstrated avid uptake in the left obturator externus muscle, the muscles of the medial and posterior compartments of the thigh, and the posterior and peroneal muscles of the calf (maximum standard uptake value [SUV_max_] = 10.2) (Fig. 7). Enlarged lymph nodes with increased FDG-uptake were observed in the left inguinal region. Bone marrow biopsy was normal, showing no evidence of lymphoma.

**Fig. 8:**
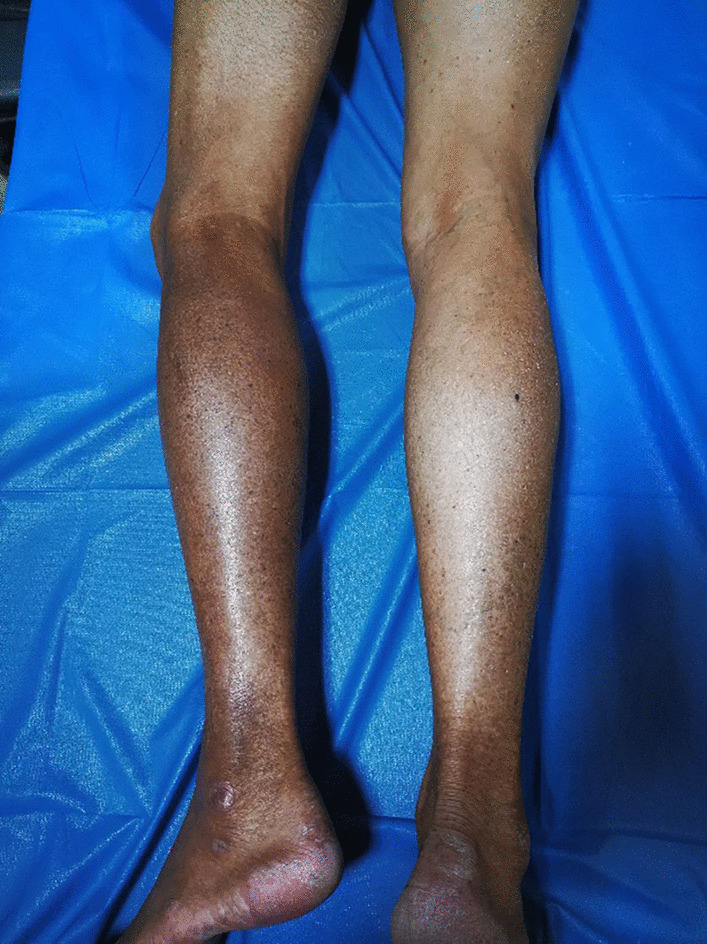
The swelling in the left lower extremity was completely resolved, but the skin remained pigmented

**Fig. 9:**
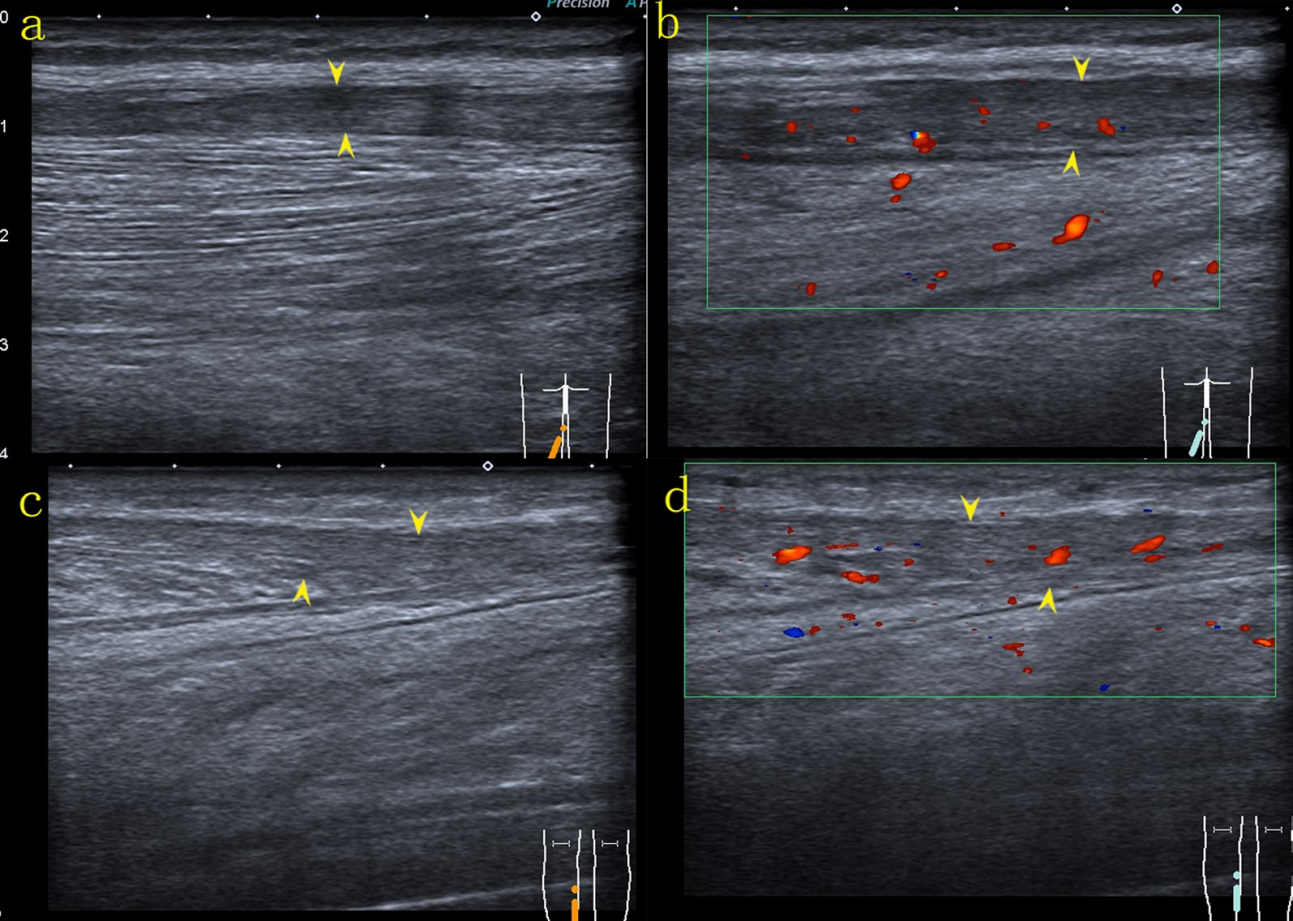
Ultrasound revealed obvious regression of the lesions, and the adductor muscles of the left thigh (**a**, **b**) and the gastrocnemius (**c**, **d**) were clearly defined with decreased peripheral echogenicity (*arrow head*). On color Doppler ultrasound, the vascularity of the affected muscles was decreased compared to the initial examination (**b**, **d**)

The patient was referred to the Haematology Department for chemotherapy where he was started on rituximab, cyclophosphamide, doxorubicin, vincristine, and prednisone (R-CHOP). To date, the patient has received four cycles (21 days each) of R-CHOP according to the following protocol: Day 1, 600 mg rituximab qd (quaque die) (intravenous pump); Days 2–4, 700 mg cyclophosphamide qd (intravenous drip); Day 2, 20 ml doxorubicin qd (intravenous drip); Day 2, 5 mg vincristine qd (intravenous drip); Days 2–6: 15 mg dexamethasone qd (intravenous drip). The patient achieved a satisfactory initial response, and the swelling of the left lower extremity gradually subsided. After the fourth cycle of R-CHOP, the swelling in the left lower extremity was completely resolved, but the skin remained pigmented (Fig. 8). US revealed obvious regression of the hypoechoic regions in the muscles of the medial and posterior compartments of the left thigh and the gastrocnemius, and on Color Doppler US, vascularity was decreased compared to the initial examination (Fig. 9). At the end of treatment, the patient underwent another US examination, which showed the size of the hypoechoic regions had decreased and the vascularity of the affected muscles was substantially reduced and only slightly greater than normal peripheral muscles. The patient is receiving regular follow-up. Eighteen months after the initial presentation in August 2019, the patient was considered clinically well and showed no signs or symptoms of lymphoma.

## Discussion and conclusions

In this report, we present a rare case of skeletal muscle DLBCL occurring in the lower extremity of a patient who had not experienced trauma or infection but who was positive for EBER. Evidence suggests that the majority of skeletal muscle lymphomas are non-Hodgkin B-cell tumours [8], with DLBCL as the most common extranodal manifestation of lymphoma involving skeletal muscle. The diagnosis of DLBCL requires multiple tests, including immunohistochemical studies, fluorescent in situ hybridization, polymerase chain reaction techniques, and occasionally, next generation sequencing [9]. Immunohistochemical staining for MYC, Bcl-2 and Bcl-6 may predict and guide treatment options in DLBCL [9]. Skeletal muscle DLBCL is frequently located in the lower extremities, especially the thigh [10–12], and usually occurs after leg injury or needle injections [12, 13]. EBV is a risk factor associated with a variety of tumours, including B and T-cell lymphomas and smooth muscle tumors [13]. Based on immunophenotype, our case is best classified as an EBV-positive DLBCL.

Skeletal muscle lymphoma is rare, with only a few cases described in the published literature. Skeletal muscle lymphoma may be primary extranodal or secondary to invasion from adjacent bone or lymph nodes, metastasis from disseminated disease, or primary extra-nodal muscle lymphoma [14, 15]. Skeletal muscle lymphoma usually presents as a painful unilateral limb swelling, representing a diagnostic challenge when differentiating muscle lymphoma from deep vein thrombosis or soft tissue tumors such as sarcoma, rhabdomyosarcoma, rhabdomyoblastoma, melanoma, and osteosarcoma [16]. Accurate diagnosis of skeletal muscle lymphoma is necessary to avoid unnecessary excisions when other entities are suspected.

Imaging tools play a critical role in the diagnosis of skeletal muscle lymphoma. On radiologic examination, skeletal muscle lymphoma appear as focal intramuscular masses and diffuse muscle enlargement with tumor infiltration [3]. Muscle enlargement is the most common pattern [3]; however, there are no specific radiologic criteria for diagnosing skeletal muscle lymphoma.

**Fig. 10:**
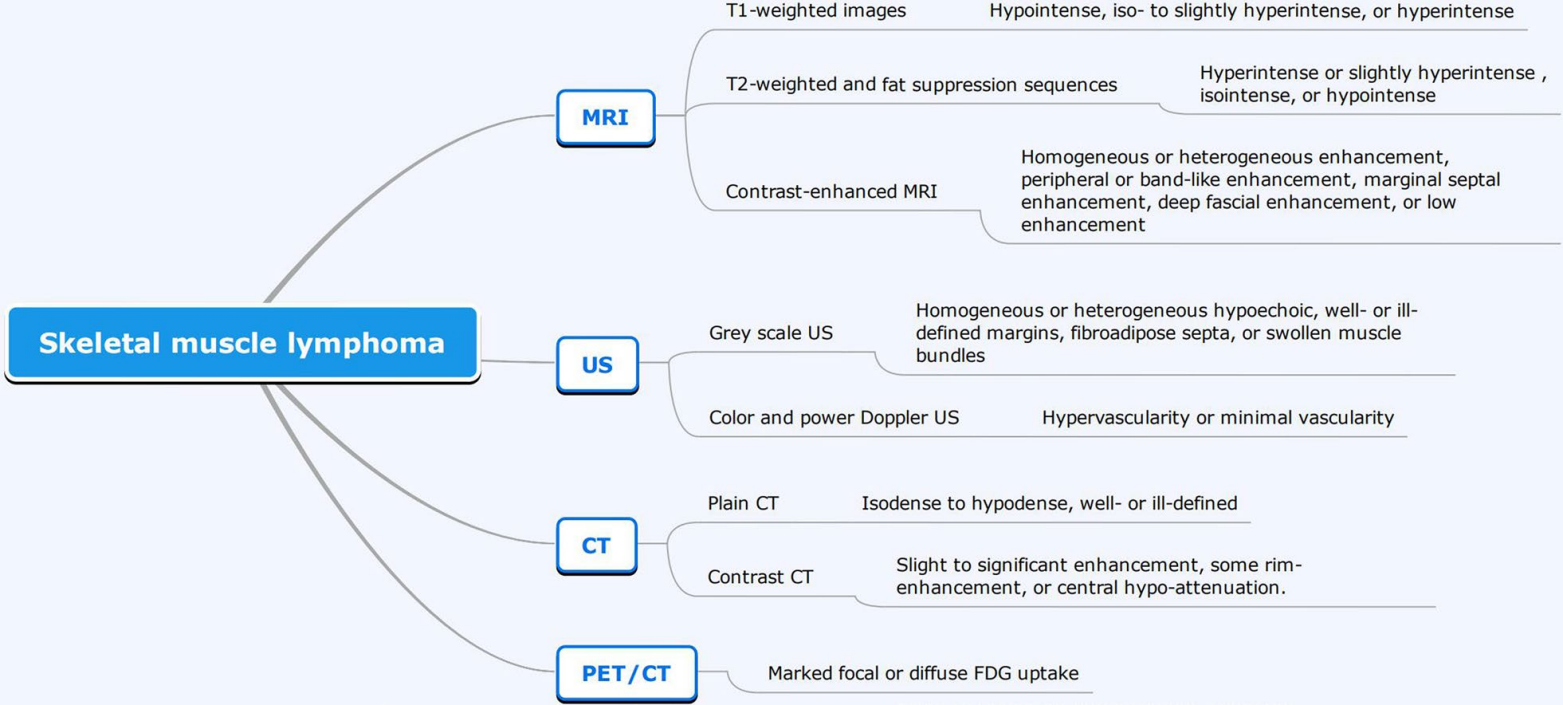
Imaging features of skeletal muscle lymphoma

To further investigate the role of imaging in the definitive diagnosis of skeletal muscle lymphoma, a systematic review of the literature published between 1992 and 2019 was conducted. The PubMed database was searched using the Mesh terms: “lymphoma”, “muscular”, “skeletal”, “soft tissue”, and “image.” Relevant case reports and case series, reviews, and retrospective studies describing skeletal muscle lymphoma were retrieved. Studies reporting on patients with a history of lymphoma at another site, skeletal muscle lymphoma arising from adjacent tumors, other systemic disease such as HIV infection, rhabdomyolysis or sarcoidosis or other organ involvement, studies with no detailed description of the radiologic features of skeletal muscle lymphoma, or literature reviews, were excluded. Searches were restricted to the English language. Finally, 25 full-text articles were reviewed, and the imaging features of 95 patients with skeletal muscle lymphoma on US, MRI (non- or contrast), CT (non- or contrast) and PET/CT, including those described for the present case, are summarized in Table 1 and Fig. 10, Findings showed the imaging features of skeletal muscle lymphoma included enlargement of muscular structures, with preservation of the architecture of the tissue and surrounding anatomical structures. Most cases were finally diagnosed by imaging-guided biopsy. To our knowledge, this is the most extensive review of the imaging features of skeletal muscle lymphoma currently available in the published literature.

**Table 1:** Published studies describing the imaging features of extranodal lymphoma of muscle (listed chronologically)

Literature	General information		US			MRI					CT			PET/CT (N)
Author/year	(Sex, N)/age	Number of affected muscles (N)	Boundary (N)	Echogenicity (N)	Vascularity (N)	Boundary (N)	T1W (N)	T1W + Gd (N)	T2W (N)	STIR (N)	Boundary (N)	Plain (N)	Contrast (N)	
Metzler et al. [23]	F/65	Multiple	–	–	–	N/A	Hypointense	No enhancement	Hypointense	Hyperintense	–	–	–	–
Eustace et al. [34]	(F, 2)/(67,68)	Single	–	–	–	Well-defined (1), N/A (1)	Isointense	Diffuse enhancement (1), N/A (1)	Hyperintense	Hyperintense (1), N/A (1)	–	–	–	–
Panicek et al. [30]	(M, 2)/(68,77)	Multiple (1), single (1)	–	–	–	N/A	N/A	N/A	N/A	N/A	N/A	Isodense to hypodense	Moderate enhancement	–
Beggs [11]	(M, 4; F, 2)/(31–76)	Multiple (5), single (1)	Ill- (3), well- (2) defined, N/A (1)	Hypoechoic (4), fibroadipose septa and swollen muscle bundles (1), patchy distal acoustic enhancement (1), N/A (1)	N/A	Ill- (3), well- (2) defined, N/A(1)	Minimally hyperintense (3), isointense (1), N/A (2)	Heterogeneous (1), diffuse (1), patchy (1) enhancement, N/A (3)	Hyperintense (4), N/A (2)	Hyperintense (2), N/A (4)	Ill- (1), well- (1) defined, N/A(4)	Isodense	Isodense (1), N/A (5)	–
Lee et al. [5]	(M, 2; F, 3)/(15–80)	Multiple (2), single (3)	–	–	–	N/A	Isointense	N/A	Hyperintense	N/A	N/A	Isodense to hypodense (2), N/A (3)	N/A	Increased uptake (2), N/A (3)
Suresh et al. [15]	(M, 14; F, 10)/(15–80)	Multiple (12), single (12)	–	–	–	Ill- (16) or well- (8) defined	Hyperintense (15), isointense (8), N/A (1)	Homogeneous or heterogeneous enhancement	Hyperintense (1), isointense (21), N/A (2)	Hyperintense (16), N/A (8)	–	–	–	–
Laffosse et al. [14]	F/66	Single	N/A	Hypoechoic	N/A	N/A	Isointense	Enhancement	N/A	N/A	–	–	–	–
Wu et al. [35]	M/14	Multiple	N/A	Hypoechoic	N/A	N/A	N/A	Enhancement	Markedly hyperintense	N/A	Ill-defined	N/A	N/A	–
Driss et al. [20]	M/8	Multiple	–	–	–	N/A	Hypointense	N/A	Hyperintense	N/A	N/A	N/A	N/A	–
Broski et al. [36]	M/65	Multiple	–	–	–	N/A	N/A	N/A	N/A	N/A	–	–	–	Increased uptake
Chun et al. [22]	(M, 14; F, 6)/(5–90)	Multiple (14), single (6)	–	–	–	N/A	Intermediate (11), hyperintense (9)	Diffuse (13), peripheral band (4), marginal septal (2), enhancement, N/A (1)	Isointense	Hyperintense (5), isointense (8), N/A (7)	–	–	–	–
Gaiser et al. [37]	M/10	Single	–	–	–	Ill-defined	N/A	Vivid enhancement	N/A	N/A	–	–	–	–
Muralee Mohan et al. [26]	M/55	Single	Ill-defined	Hypoechoic, muscle like texture	Minimal	N/A	Isointense	N/A	Heterogeneously hyperintense	Hyperintense	Well-defined	Isodense	Homogeneous enhancement	–
Carroll et al. [18]	(M, 2; F, 5)/(56–68)	Multiple (2), single (5)	–	–	–	Ill- (3) or well- (4) defined	Iso- to slightly hyperintense	Homogeneous enhancement	Homogeneously or heterogeneously hyperintense	N/A	–	–	–	–
Hongsakul et al. [38]	F/45	Multiple	Ill-defined	Hypoechoic, fibroadipose septa and swollen muscle bundles	N/A	N/A	Slightly hyperintense	Inhomogeneous enhancement	Slightly hyperintense	N/A	N/A	Enlargement	Enhancement	–
Surov [3]	(M, 4; F, 6)/(45–75)	Multiple (1), single (9)	–	–	–	N/A	Homogeneously hypointense	N/A	Hyperintense	Hyperintense	–	–	–	–
Katsura et al. [33]	F/52	Multiple	–	–	–	–	–	–	–	–	N/A	N/A	Non-uniformly early enhancing, central necrosis	Increased uptake
Zhang et al. [39]	F/76	Multiple	N/A	Hypoechoic	N/A	N/A	N/A	Enhancement	N/A	N/A	–	–	–	–
Hatem et al. [32]	M/70	Single	Ill-defined	Hypoechoic	N/A	Ill-defined	N/A	N/A	N/A	N/A	–	–	–	Increased uptake
Elkourashy et al. [40]	M/40	Multiple	–	–	–	N/A	Abnormal	Heterogeneous enhancement	N/A	N/A	–	–	–	–
Burton et al. [41]	M/17	Multiple	–	–	–	N/A	Isointense	Diffuse enhancement	Diffusely mildly hyperintense	N/A	–	–	–	–
Spetsieris et al. [29]	F/70	Multiple	N/A	Hypoechoic	Increased	N/A	Hypointense	Enhancement	Hyperintense	N/A	N/A	Isodense	N/A	–
Martins et al. [42]	M/76	Multiple	–	–	–	Well-defined	Isointense	Peripheral enhancement	Hyperintense	Hyperintense	–	–	–	Increased uptake
Binici et al. [43]	F/41	Multiple	–	–	–	Ill-defined	Isointense	N/A	Hyperintense	N/A	–	–	–	–
Present case/2019	M/70	Multiple	Ill-defined	Hypoechoic, muscle fibers	Increased	Ill-defined	Minimally hyperintense	N/A	Hyperintense	N/A	Ill-defined	Hypodense	mild-moderate enhancement	Increased uptake

*T1W* T1-weighted sequence; *T2W* T2-weighted sequence; *Gd* gadolinium; *STIR* short-tau inversion recovery; *US* ultrasound; *MRI* magnetic resonance imaging; *CT* computed tomography; *PET/CT* positron emission tomography/computed tomography; *N/A* not available

N refers to the number of patients

MRI has been considered the most useful modality for assessment of skeletal muscle lymphoma [17]; however, MRI is costly, patients may have contraindications, and MRI characteristics of skeletal muscle lymphoma are nonspecific and sometimes contradictory. Studies have reported skeletal muscle lymphoma appear hypointense, isointense, or slightly hyperintense relative to surrounding muscle on T1-weighted images and mostly hyperintense on T2-weighted and fat suppression sequences [5, 18–21]. Contrast-enhanced MRI has shown diffuse homogeneous or heterogeneous enhancement [5, 11], peripheral thick band-like enhancement, marginal septal enhancement, or deep fascial enhancement [17, 22], which may have been caused by multicompartment infiltration [22]. One report [23] described a case of skeletal muscle lymphoma with no contrast enhancement in any of the affected muscles. Several features on MRI are suggestive of skeletal muscle lymphoma and may serve to differentiate skeletal muscle lymphoma from other intramuscular malignant tumours. These include infiltration of the subcutaneous fat with occasional skin thickening or edema [5, 11, 15, 22], although this was not observed in the present case; multicompartment muscle involvement [5, 15, 24]; tumor extension along neurovascular bundles or muscle fascicles; and vessels traversing the affected muscles [15, 17].

US is helpful for delineating soft tissue lesions. On US, skeletal muscle lymphoma may be suspected in cases showing diffuse muscle enlargement with multicompartment muscle involvement and swelling of muscle bundles. Skeletal muscle lymphoma may appear as homogeneous or heterogeneous hypoechoic masses with lobulated, irregular, well or poorly defined margins and coarsened fibroadipose septa [3, 8, 11, 17, 25, 26], or hypoechoic regions with texture resembling muscle fibers retaining continuity with the surrounding muscles, as in the present case and one other previous study [26]. This feature has not been described in other intramuscular lesions such as sarcoma, myositis and inflammatory pseudotumors, and may be an important consideration for differential diagnosis. This feature may reflect the degree of muscle involvement [11, 27] and correlate with histological findings that show neoplastic lymphocytes surrounding and invading myofibrils [27, 28]. Color and power Doppler US may show hypervascularity [25, 29]. Real-time US-guided core needle biopsy is often used for diagnosis of skeletal muscle lymphoma, and was applied in the present case.

CT is frequently performed for initial evaluation of soft-tissue masses in patients for whom MRI may be contraindicated due to the presence of intracranial aneurysm clips, a cardiac pacemaker, or a predisposition to claustrophobia [25, 30]. CT characteristics of skeletal muscle lymphoma are nonspecific. On plain CT, skeletal muscle lymphoma may appear as swelling of the affected muscles with well- or ill-defined lesions that are homogeneous, isodense, hyperdense or hypodense to normal muscle [3, 5, 11, 25, 31]. On contrast CT, skeletal muscle lymphoma may show slight to significant enhancement [5, 11], some rim-enhancement, or central hypo-attenuation [31]. Other common malignancies of soft tissue, including malignant fibrous histiocytoma, fibrosarcoma and liposarcoma, also appear as heterogeneous masses with variable enhancement on CT [30] and must be differentiated from skeletal muscle lymphoma. Skeletal muscle lymphoma may be suspected where CT shows a homogeneous mass with attenuation similar to that of normal muscle and traversing vessels in the affected muscle [30], as in the present case.

PET/CT fusion is an important imaging tool for initial staging, assessment of treatment efficacy, and restaging after treatment in patients with cancer [31]. In skeletal muscle lymphoma, there is usually marked focal or diffuse FDG uptake in the affected muscles, and uptake levels may be correlated with malignant potential; however, it is difficult to differentiate primary or secondary skeletal muscle lymphoma from other malignant tumours and inflammatory lesions. In the present case, PET/CT imaging showed increased FDG uptake in multiple muscles of the left thigh and calf.

The workup of patients with skeletal muscle lymphoma should include imaging, image-guided biopsy, histological examination of the biopsy to confirm the diagnosis, and clinical staging to select appropriate treatment. The combination of chemotherapy and immunotherapy (R-CHOP) with or without radiation therapy is considered standard therapy for DLBCL [32]. This approach achieves a good response in patients with skeletal muscle lymphoma [29, 32, 33]. However, a meta analysis [24] revealed that DLBCL of the soft tissue is aggressive and associated with poor prognosis, requiring further studies to better characterize this peculiar entity.

In conclusion, lymphoma arising in the skeletal muscle is rare. The majority of skeletal muscle lymphomas are B cell lymphomas, especially DLBCL. US, CT, and MRI can be used to identify muscle lymphoma. On US, skeletal muscle lymphoma should be suspected in patients presenting with lesions that appear as hypoechoic masses containing residual muscle fibers or swollen muscle bundles and that involve multiple muscles. The definitive diagnosis of skeletal muscle lymphoma relies on histological and immunohistological analysis of a sample obtained through imaging-guided biopsy and proper staging, which inform clinical decision making.

## Data Availability

The datasets generated and analyzed during the present study are available from the corresponding author on reasonable request.

## Abbreviations

US: Ultrasound
CT: Computed tomography
MRI: Magnetic resonance imaging
CA125: Cancer antigen 125
IHC: Immunohistochemical
ESR: Erythrocyte sedimentation rate determination
CRP: C-reaction protein
B2-MG: B2 mircoglobulin
LDH: Lactate dehydrogenase
